# Hydrogenated Amorphous Silicon Charge-Selective Contact Devices on a Polyimide Flexible Substrate for Dosimetry and Beam Flux Measurements

**DOI:** 10.3390/s25041263

**Published:** 2025-02-19

**Authors:** Mauro Menichelli, Saba Aziz, Aishah Bashiri, Marco Bizzarri, Clarissa Buti, Lucio Calcagnile, Daniela Calvo, Mirco Caprai, Domenico Caputo, Anna Paola Caricato, Roberto Catalano, Massimo Cazzanelli, Roberto Cirio, Giuseppe Antonio Pablo Cirrone, Federico Cittadini, Tommaso Croci, Giacomo Cuttone, Giampiero de Cesare, Paolo De Remigis, Sylvain Dunand, Michele Fabi, Luca Frontini, Catia Grimani, Mariacristina Guarrera, Hamza Hasnaoui, Maria Ionica, Keida Kanxheri, Matthew Large, Francesca Lenta, Valentino Liberali, Nicola Lovecchio, Maurizio Martino, Giuseppe Maruccio, Giovanni Mazza, Anna Grazia Monteduro, Arianna Morozzi, Augusto Nascetti, Stefania Pallotta, Andrea Papi, Daniele Passeri, Maddalena Pedio, Marco Petasecca, Giada Petringa, Francesca Peverini, Pisana Placidi, Matteo Polo, Alberto Quaranta, Gianluca Quarta, Silvia Rizzato, Federico Sabbatini, Leonello Servoli, Alberto Stabile, Cinzia Talamonti, Jonathan Emanuel Thomet, Luca Tosti, Monica Setia Vasquez Mora, Mattia Villani, Richard James Wheadon, Nicolas Wyrsch, Nicola Zema

**Affiliations:** 1 INFN, Sezione di Perugia, Via Pascoli s.n.c., 06123 Perugia, Italy; marco.bizzarri@unipg.it (M.B.); mirco.caprai@pg.infn.it (M.C.); federico.cittadini@phd.unipd.it (F.C.); tommaso.croci@pg.infn.it (T.C.); maria.ionica@pg.infn.it (M.I.); keida.kanxheri@pg.infn.it (K.K.); arianna.morozzi@pg.infn.it (A.M.); andrea.papi@pg.infn.it (A.P.); daniele.passeri@unipg.it (D.P.); pedio@iom.cnr.it (M.P.); francesca.peverini@pg.infn.it (F.P.); pisana.placidi@unipg.it (P.P.); leonello.servoli@pg.infn.it (L.S.); nicola.zema@ism.cnr.it (N.Z.); 2 INFN Sezione di Lecce, Dipartimento di Fisica e Matematica, dell’Università del Salento, Via per Arnesano, 73100 Lecce, Italy; saba.aziz@unisalento.it (S.A.); lucio.calcagnile@unisalento.it (L.C.); annapaola.caricato@unisalento.it (A.P.C.); maurizio.martino@unisalento.it (M.M.); giuseppe.maruccio@unisalento.it (G.M.); annagrazia.monteduro@unisalento.it (A.G.M.); gianluca.quarta@unisalento.it (G.Q.); silvia.rizzato@unisalento.it (S.R.); 3 Centre for Medical Radiation Physics, University of Wollongong, Northfields Ave Wollongong, NSW 2522, Australia; amab916@uowmail.edu.au (A.B.); mjl970@uowmail.edu.au (M.L.); marcop@uow.edu.au (M.P.); 4 Physics Department, Faculty of Science and Art, Najran University, King Abdulaziz Rd,1988 Najran, Saudi Arabia; 5 Dipipartimento di Fisica e Geologia, dell’Università degli Studi di Perugia, Via Pascoli s.n.c., 06123 Perugia, Italy; 6 INFN Sezione di Firenze, Via Sansone 1, 50019 Sesto Fiorentino, Italy; clarissa.buti@edu.unifi.it (C.B.); michele.fabi@uniurb.it (M.F.); catia.grimani@uniurb.it (C.G.); stefania.pallotta@unifi.it (S.P.);federico.sabbatini@uniurb.it (F.S.); cinzia.talamonti@unifi.it (C.T.); mattia.villani@uniurb.it (M.V.); 7 Department of Experimental and Clinical Biomedical Sciences “Mario Serio”, University of Florence, Viale Morgagni 50, 50135 Firenze, Italy; 8 INFN Sezione di Torino Via Pietro Giuria, 110125 Torino, Italy; calvo@to.infn.it (D.C.); cirio@to.infn.it (R.C.); deremigi@to.infn.it (P.D.R.); francesca.lenta@polito.it (F.L.); mazza@to.infn.it (G.M.); wheadon@to.infn.it (R.J.W.); 9 INFN Sezione di Roma 1, Piazzale Aldo Moro 2, 00185 Roma, Italy; domenico.caputo@uniroma1.it (D.C.); giampiero.decesare@uniroma1.it (G.d.C.); nicola.lovecchio@uniroma1.it (N.L.); augusto.nascetti@uniroma1.it (A.N.); 10 Dipartimento Ingegneria dell’Informazione, Elettronica e Telecomunicazioni, dell’Università degli studi di Roma, Via Eudossiana, 18, 00184 Roma, Italy; 11 INFN Laboratori Nazionali del Sud, Via S.Sofia62, 95123 Catania, Italy; catalano@lns.infn.it (R.C.); cirrone@lns.infn.it (G.A.P.C.); cuttone@lns.infn.it (G.C.); guarrera@lns.infn.it (M.G.); petringa@lns.infn.it (G.P.); 12 Dipatimento di Ingegneria, TIFPA and Trento University, Via Sommarive 14, 38123 Povo, Italy; massimo.cazzanelli@unitn.it (M.C.); hamza.hasnaoui@unitn.it (H.H.); polo.matteo@unitn.it (M.P.); alberto.quaranta@unitn.it (A.Q.); 13 Dipatimento Di Fisica e Astronomia, dell’Università di Padova, Via Marzolo 8, 35131 Padova, Italy; 14 Dipatimento di Ingegneria, dell’Università degli studi di Perugia, Via G.Duranti, 06125 Perugia, Italy; 15 Ecole Polytechnique Fédérale de Lausanne (EPFL), Institute of Electrical and Microengineering (IME), Rue de la Maladière 71b, 2000 Neuchâtel, Switzerland; sylvain.dunand@epfl.ch (S.D.); jonathan.thomet@epfl.ch (J.E.T.); nicolas.wyrsch@epfl.ch (N.W.); 16 DiSPeA, Università di Urbino Carlo Bo, 61029 Urbino, Italy; 17 INFN Sezione di Milano Via Celoria 16, 20133 Milano, Italy; luca.frontini@unimi.it (L.F.); valentino.liberali@mi.infn.it (V.L.); alberto.stabile@mi.infn.it (A.S.); monica.vasquez@mi.infn.it (M.S.V.M.); 18 Dipartimento di Fisica, dell’Università degli Studi di Milano, Via Celoria 16, 20133 Milano, Italy; 19 Politecnico di Torino Facoltà di Ingegneria, Corso Duca degli Abruzzi 24, 10129 Torino, Italy; 20 Scuola di Ingegneria Aerospaziale, Università degli studi di Roma, Via Salaria 851/881, 00138 Roma, Italy; 21 CNR-IOM, Via Pascoli s.n.c., 06123 Perugia, Italy; 22 CNR Istituto Struttura Della Materia, Via Fosso del Cavaliere 100, 00133 Roma, Italy

**Keywords:** dosimeters, hydrogenated amorphous silicon detectors, radiation hardness, charge-selective contacts devices, flexible sensors

## Abstract

Hydrogenated amorphous silicon (a-Si:H) devices on flexible substrates are currently being studied for application in dosimetry and beam flux measurements. The necessity of in vivo dosimetry requires thin devices with maximal transparency and flexibility. For this reason, a thin (<10 µm) a-Si:H device deposited on a thin polyimide sheet is a very valid option for this application. Furthermore, a-Si:H is a material that has an intrinsically high radiation hardness. In order to develop these devices, the HASPIDE (Hydrogenated Amorphous Silicon Pixel Detectors) collaboration has implemented two different device configurations: n-i-p type diodes and charge-selective contact devices.Charge-selective contact-based devices have been studied for solar cell applications and, recently, the above-mentioned collaboration has tested these devices for X-ray dose measurements. In this paper, the HASPIDE collaboration has studied the X-ray and proton response of charge-selective contact devices deposited on Polyimide. The linearity of the photocurrent response to X-ray versus dose-rate has been assessed at various bias voltages. The sensitivity to protons has also been studied at various bias voltages and the wide range linearity has been tested for fluxes in the range from 8.3 × 10^7^ to 2.49 × 10^10^ p/(cm^2^ s).

## 1. Introduction

Hydrogenated amorphous silicon (a-Si:H) is an amorphous semiconductor material that was originally developed back in 1969 [1]. After the first successful doping attempt, it was possible to use this material in electronics, solar cells, and radiation detectors [2,3,4]. The most used deposition technique for this material is plasma-enhanced chemical vapor deposition (PECVD) from a mixture of silane (SiH_4_) and hydrogen at temperatures of about 180–250 °C [5]. The low deposition temperature of a-Si:H allows its layering on flexible materials like polyimide (PI). The typical device structure for radiation dose measurement is the n-i-p diode structure where an n-doped layer is deposited on a metal stack formed by aluminum and chromium for backside contacts. On top of this n-doped layer, an intrinsic layer of a-Si:H where the radiation interacts with the semiconductor material generating hole-electron pairs collected by the electric field generated by an external bias is deposited [6]. On top of the intrinsic a-Si:H layer, a layer of p-doped a-Si:H and another metal layer(or an ITO—Indium Tin Oxide—layer) for the electrical contacts completes the device. Charge-selective contact (CSC) devices are similar to n-i-p devices where n-doped material is replaced by electron selective contact material (TiO_2_ in the case of the present development) and the p-type material is replaced by a hole-selective material (in the present case MoO_x_). Therefore, charge-selective contact devices [7] are based on a three-layer structure featuring a thin layer of metal-oxides with a small activation energy (like TiO_2_), a thick layer of intrinsic a-Si:H, and a thin layer of metal-oxides with a large activation energy (like MoO_x_ or WO_x_). A similar device deposited on glass has also been tested with excellent results for radiation displacement damage with neutrons up to 10^16^ n_eq_/cm^2^ [8], demonstrating that this material is sufficiently robust to withstand radiation levels typical for a radiation detector used in medical applications for a reasonably long time.

In medical applications, traditional dose control in external beam radio-therapy (EBRT), is performed before the actual treatment using standardized dose measurements obtained by ionization chambers in water-equivalent phantoms [9]. Although these standard procedures are sufficiently adequate for pre-treatment dose estimations, (a) they cannot account for differences in patient geometry between planning and treatment stages, and (b) they cannot take account of any deviations in the actual clinical dose during delivery due to any fluctuation in external conditions. For these reasons, real-time dosimetry using flexible devices is essential. As mentioned in [10] real-time dosimetry for treatments with a linear accelerator (LINAC) should be provided at four points: the collimator, the patient body surface, the couch, and finally through electronic portal imaging devices (EPIDs). This capability to provide real-time verification of the dose delivered to the patient is what is defined as in vivo dosimetry. In vivo dosimetry is rapidly becoming a compulsory component of dose monitoring practices for EBRT.

In many European countries, in vivo dosimetry is now mandatory according to the Medical Exposure Directive (MED)97/43/Euratom [11]. In vivo dosimetry has clear advantages in treatment modalities where the treatment planning may have serious uncertainties, like when irradiations delivered at shallow depths and total body irradiations are foreseen. Above all, in vivo dosimetry provides an accurate means for the delivered dose verification, which can be a useful help in minimizing the risk of excessive dose delivery that may cause severe injury or even, in extreme cases, death [12]. Detectors must meet several strict requirements in order to be able to perform effectively real-time in vivo dosimetry:

- The ability, with a very high level of accuracy, to measure skin dose or dose in the build-up region.
- The right flexibility as to conform to the patient’s treatment area.
- Angular independent response.
- Good transparency to provide minimal perturbation to the treatment-beam profile and intensity.

Different technologies are adopted to provide solutions to the challenges mentioned above. Radiochromic films are flexible and provide excellent tissue equivalence, spatial resolution, and the ability to perform dosimetry over a large area [13]. However, these dosimetric techniques do not provide real-time measurements of the dose impinging the patient’s body. Concerning real-time large-area dosimetry, several solutions are available based solely on EPID or a combination of EPID and dedicated detectors. These large-area pixelated detectors typically employ a 2D array of silicon detectors placed on the flat panel imager available on most clinical LINACs to monitor exit dose during patient treatments. Unfortunately, these EPIDs have complex tissue-equivalent correction factors, due to the high-Z semiconductor materials typically employed in fabricating EPIDs, MOSFETs, and other solid-state dosimeters.

Furthermore, the commercially available EPIDs are not flexible enough to conform to the patient’s body surface. Fiber optic and organic semiconductor detectors provide both flexibility and adequate tissue-equivalence. However, both technologies are based on the indirect detection of X-rays performed by scintillators. These scintillators can generate parasitic signals due to photoluminescence or Cerenkov radiation, which very often result in a reduction in the spatial resolution and loss of dosimetric accuracy [14,15,16]. Modern radio-luminescent dosimeters, point-like or multi-point-like for in vivo dosimetry, are able to mitigate or to apply correction factors to take account for these limitations, resulting in accurate full-field in vivo dosimetry from scintillator-based detectors [17,18]. In summary, there is an actual necessity for new detector technologies providing accurate real-time patient dosimetry, a sufficiently large area, tissue equivalency, flexibility, good spatial resolution, and transparency to radiation in order to properly address all the challenges offered by advanced treatment modalities. For these reasons, these a-Si:H devices on polyimide seem to adequately meet all these goals. Samples having n-i-p structure have already been characterized for this purpose by the HASPIDE collaboration [19].

In the present paper, the X-ray response of samples having charge selective contacts will be shown. The tests described here include the following: dark current measurements at various bias voltages, the photocurrent versus X-ray dose-rate curve and X-ray dose sensitivity at various bias voltages, sensitivity to protons, and linearity of response with protons in a 2.5 order of magnitude range.

## 2. Materials and Methods

**Figure 1:**
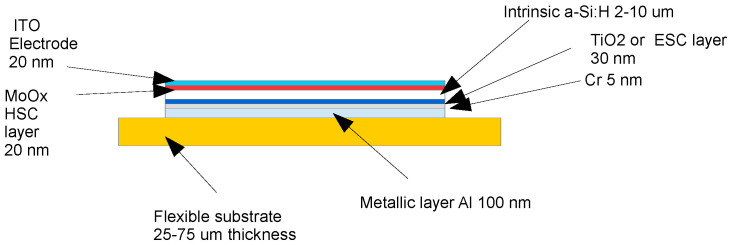
Layout of HASPIDE charge-selective diode prototype. HSC stands for hole-selective contact and ESC stands for electron-selective contact.

**Figure 2:**
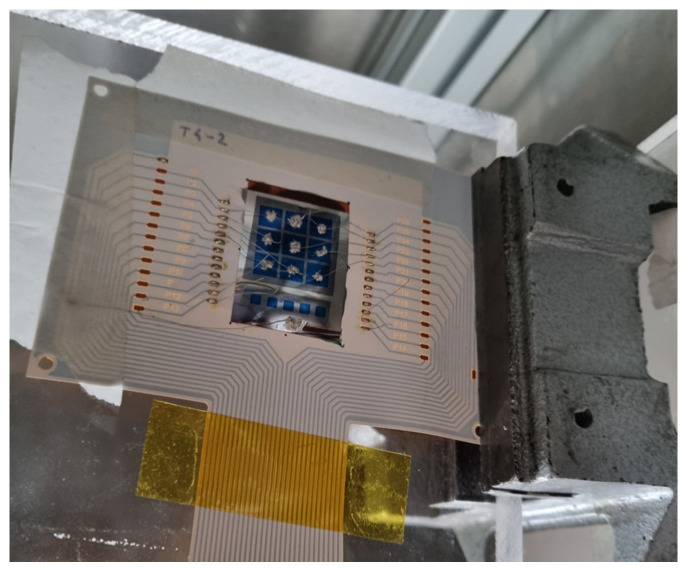
Picture of the detector array bonded on the PI PCB. Nine 5 mm × 5 mm devices and five 2 mm × 2 mm devices are deposited on Polyimide. The blue areas correspond to the top ITO-covered hole-selective contacts (HSC)bonded to different pads for bias and current readout, the gray area is the intrinsic a-Si:H and the shiny gray area on the bottom side of the sample is the Al/Cr back-contact bonded to the PCB and connected to ground.

The detailed structure of a CSC device is shown in Figure 1. On the top of a 25 µm thick Polyimide substrate, an aluminum layer is deposited via sputtering (100 nm thickness) for the backside contact, that, in the measurements described in this paper, is grounded. In order to avoid diffusion of Aluminum in the upper layers, a layer of 5 nm of chromium is deposited over the aluminum using the same technique. On top of this metal, a layer of electron-selective contact material (namely TiO_2_) is deposited (ca. 30 nm thickness) via sputtering. On top of this layer, a layer of intrinsic a-Si:H is deposited via PECVD (Plasma Enhanced Chemical Vapor Deposition) having thickness ranging, in our applications, from 2 to 10 µm. On top of this layer, a patterned sputtering deposition of hole-selective contact material (MoO_x_) is performed. The latter is protected by a deposition of a thin indium tin oxide (ITO) layer also performed via sputtering. A picture that shows the detector tested bonded on a polyimmide (PI) PCB is shown in Figure 2.

The tested sample (Figure 2) includes five 2 mm × 2 mm and nine 5 mm × 5 mm CSC sensors with a 2.6 µm thick intrinsic a-Si:H layer and is glued and bonded (using a conductive glue) to a PI PCB. One of the 5 mm × 5 mm sensors is connected to an interface board linked to a Keithley 2400 SMU (Source Measuring Unit- now produced by Tektronix, Beaverton (OR) - USA) that is used for biasing the sensor and measuring the output current with an accuracy of 1 pA.

**Figure 3:**
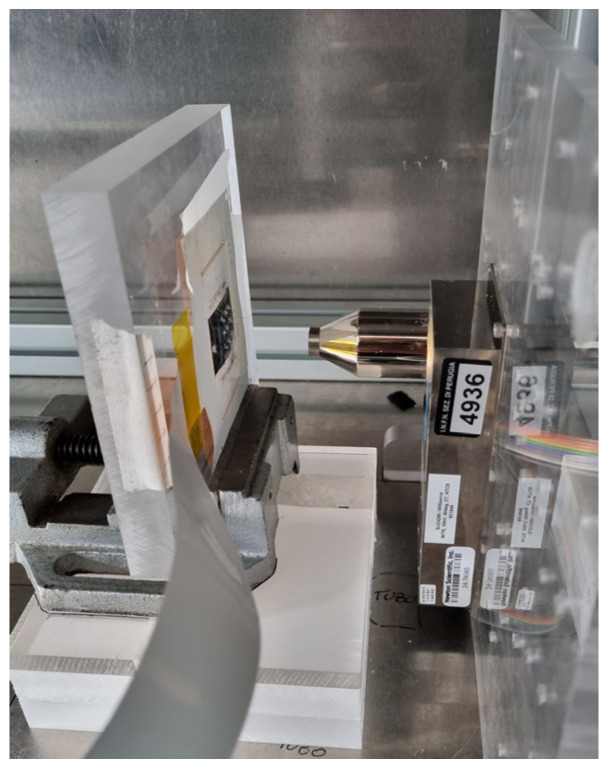
Setup for X-ray testing. The detector shown in Figure 2 is connected to the SMU through the PI PCB interface frame shown in the same figure. The picture also shows the X-ray tube. The entire setup is enclosed in a radiation-shielded cabinet.

**Figure 4:**
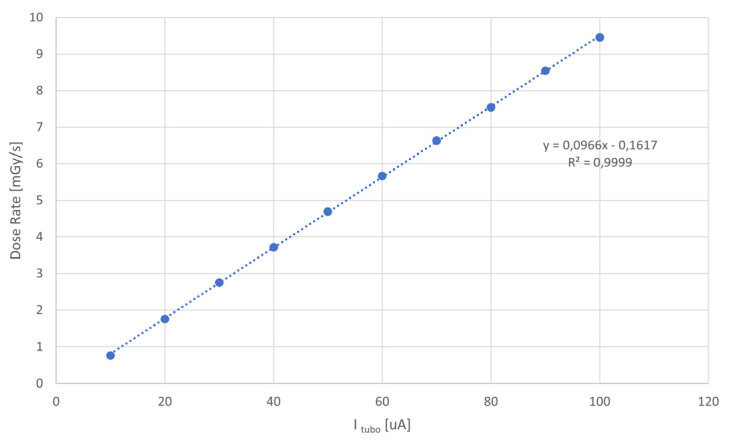
Calibration curve of dose rate versus tube current at 40 kV tube bias voltage using a dosimetric probe placed in the same position as the detector.

Figure 3 shows the irradiation setup enclosed in cabinet. The tube is a Newton Scientific M25 [20] having a maximum biasing voltage of 50 kV (5 kV minimum) emitting X-rays from a tungsten anode. The tube has an inherent filtration from a Beryllium 0.125 mm thick window. Its maximum power rating is 4 W and maximum current is 200 µA. The dose rate versus tube current at 40 kV (tube bias voltage used in this measurement) has been measured by placing the dosimeter probe (T20), read by a COBIA FLEX dosimeter from RTI [21], in the place of the detector, establishing a correspondence between dose rate and tube current. The resulting calibration curve is shown in Figure 4.

The signal from the sensor is obtained by subtracting the dark current to the current measured when the detector is illumined by X-rays at a given dose rate. The charge in the sensor is generated by ionization due to a photoelectric effect in the intrinsic layer of the detector. This charge drifts inside the intrinsic layer (diffusion is negligeable) due to the internal electric field generated by the charge-selective contact material in addition to the external bias voltage; some of this charge is captured and trapped by mid-gap defects typical of the amorphous material, this causes some charge collection inefficiency. This drift-based charge collection current which constitutes the actual signal superposes with dark current mostly due to thermal effects that need to be subtracted from the total current.

**Figure 5:**
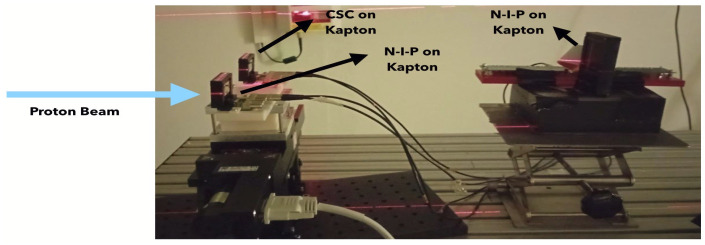
The test setup in Trento Hospital proton accelerator. A CSC and a n-i-p detector were placed on a motorized stage that allows each device to be exposed to the beam. A further n-i-p device was placed downstream of the beam.

Charge-selective contact devices have also been tested at the Trento Hospital proton accelerator in conjunction with n-i-p devices, and the results for n-i-p devices will be described in a forthcoming paper. The Trento Hospital accelerator is a proton cyclotron produced by the IBA company [22](Ion Beam Applications, Louvain-La-Neuve, Belgium) capable of accelerating protons in an energy range from 70 to 228 MeV with intensities ranging from 1 nA to 320 nA. The beam spot has a Gaussian shape, having σ_x_ and σ_y_ ranging from 2.7 to 6.9 mm depending on the energy; further details on this accelerator can be found in ref. [23]. The test setup is shown in Figure 5. The detectors (both CSC and n-i-p) were placed in a motorized stage that allowed to place in the beamline either a CSC device or a n-i-p. In the beamline, downstream of these two detectors, another n-i-p sensor was placed for characterization. The beam spot had a diameter of 9 mm and there were no scatterer (just 1.25 m of air) and no raster-scanning was used.

## 3. Results

Charge-selective contact devices have been tested for leakage current, X-rays sensitivity and linearity of response to protons (145 MeV of energy) at the Trento proton accelerator facility on a wide range of fluxes spanning 2.5 order of magnitude.

### 3.1. Leakage Current Testing

**Figure 6:**
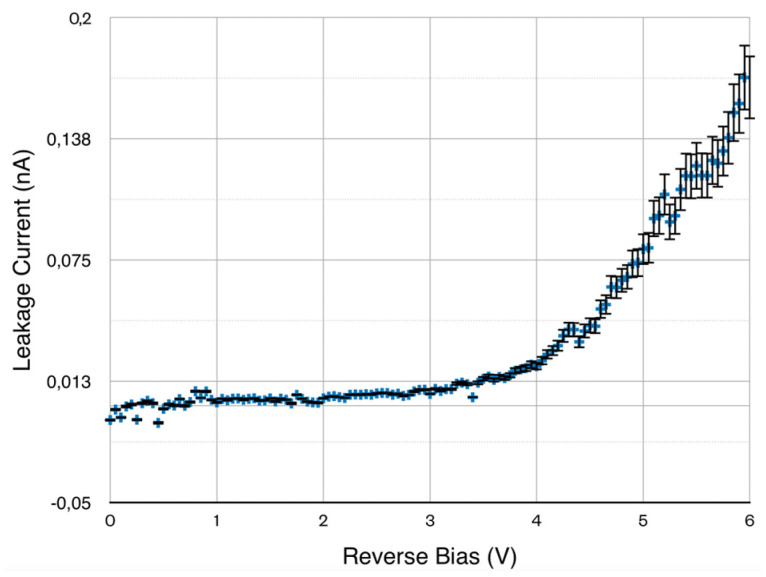
Leakage current vs. bias voltage of a 5 × 5 mm^2^charge-selective contact device.

A 5 mm × 5 mm charge-selective contact device has been tested and characterized with X-rays. Figure 6 shows the dark current versus bias voltage. The dark current is very small (below 0.025 nA)below 4 V of bias; above that bias voltage shows an exponential rise reaching about 0.175 nA at 6V.

### 3.2. X-Ray Response Testing

**Figure 7:**
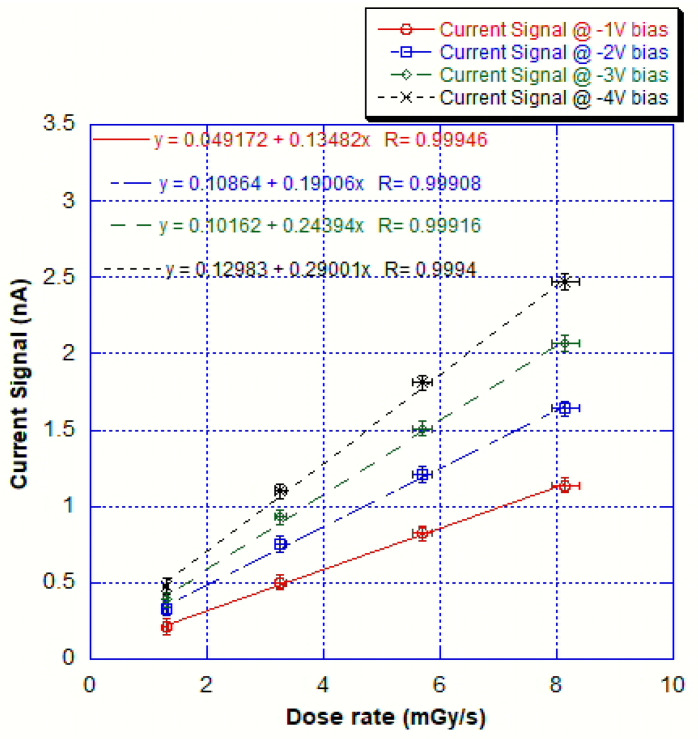
Net photocurrent versus incident X-ray dose rate for the tested device at various bias voltages.

**Figure 8:**
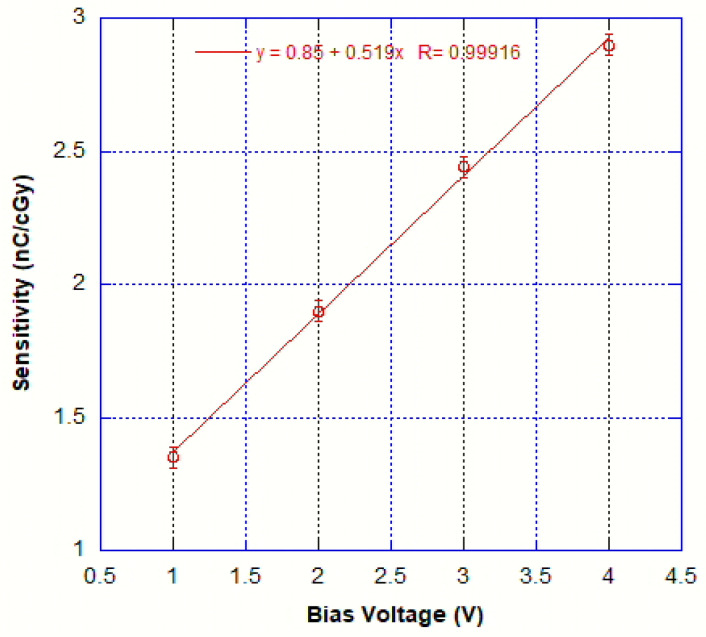
Sensitivity (nC/cGy) versus bias voltages for the tested device.

In order to measure X-ray responses at various dose rates and various detector bias voltages in the range 1–4 V (i.e., before the background current rise) using the tube mentioned in the previous section, the dose rate of the emitted radiation in this setup was measured according to the procedure shown in the previous section. After the subtraction of the leakage current, the photocurrent has been measured versus X-ray tube emitted dose rate at various device bias voltages and the results are plotted in Figure 7.The results show signals much above the dark current values at dose rates of about 1.2 mGy/s and a very linear behavior (with R parameters ranging from 0.99908 to 0.99946). The dosimetric sensitivities and linear regression coefficients have been extracted from the slopes of the linear fits and these are shown in Figure 8. We noted a linear increase in dosimetric sensitivities versus device bias voltage.

### 3.3. Proton Response Testing

**Figure 9:**
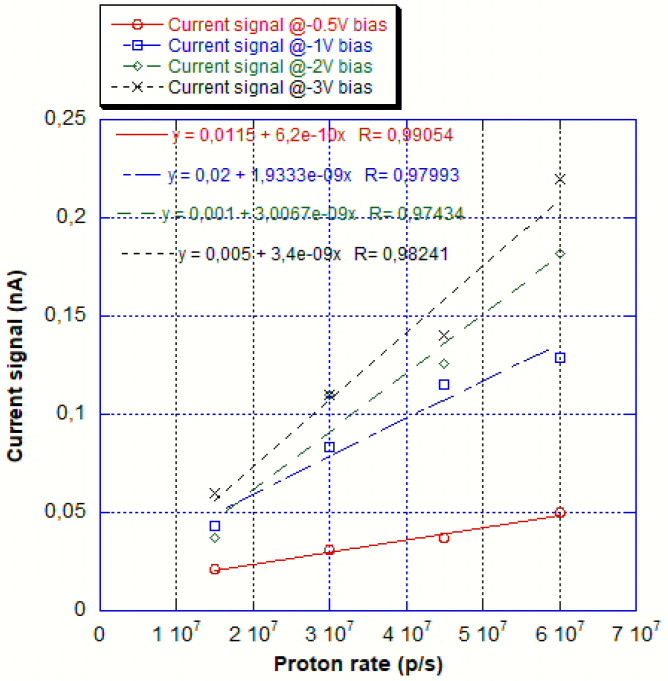
Sensitivity to proton test results. The measurement has been repeated with 4 different bias volume with results ranging from 6.2 × 10^−10^ to 3.4 × 10^−9^nC/p. The linearity in response is reasonably good.

As mentioned in the previous section, a CSC device has also been tested for sensitivity to proton and for linearity on a very wide range of fluxes. For the sensitivity measurement, the device was irradiated with four different fluxes in the range from 10^7^ p/s to 6 × 10^7^ p/s and the test has been repeated for four bias voltages: 0.5, 1.5, 2, and 3 V. The results are shown in Figure 9. The linearity is quite good for all biasing voltages, while the sensitivities range from 6.2 × 10^−10^ to 3.4 × 10^−9^nC/p. 

**Figure 10:**
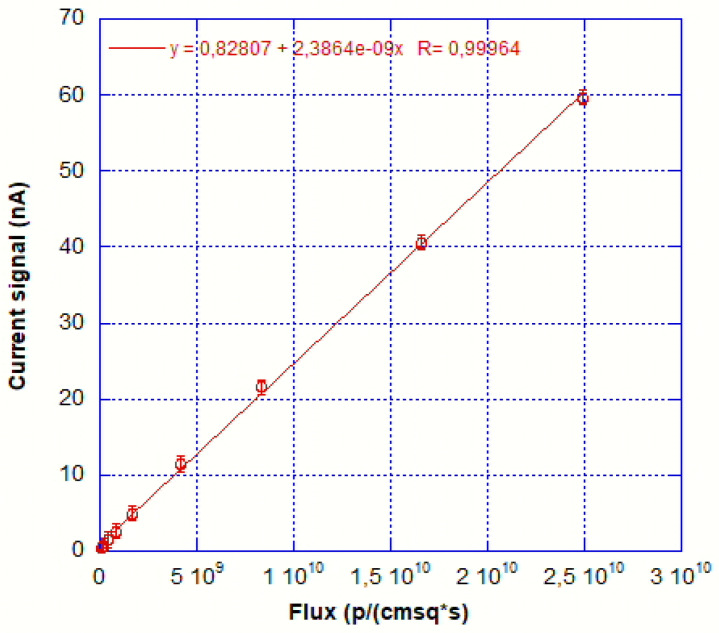
Flux versus sensor response for fluxes ranging from 8.3 × 10^7^p/(cm^2^ *s) to 2.49 × 10^10^p/(cm^2^ *s).

Concerning wide-range linearity, the device has been tested at −1V bias using a flux spanning almost 3 orders of magnitude from 8.3 × 10^7^ p/(cm^2^ *s)to 2.49 × 10^10^p/(cm^2^ *s) at 145 MeV and the linearity is impressive having an R coefficient above 0.999.The results are shown in Figure 10.

## 4. Discussion

One sample of 5 mm × 5 mm a-Si:H charge-selective contact devices on PI, having 2.6 µm thickness, have been tested in the context of the HASPIDE project, aiming at the construction of flexible planar detectors for dosimetry, radiation flux measurements, and neutron dosimetry. This device has been tested for leakage current, linearity, and dosimetric sensitivity at various bias voltages, both with protons and X-rays. The results show a very good linearity in the dose rate range tested, in addition to a good sensitivity and quite low leakage current below 4 V bias. Dosimetric sensitivity is related to bias voltage, in a very linear behavior. The response to protons have also been tested in the Trento Hospital proton accelerator, giving good results in terms of sensitivities at bias values ranging from 0.5 to 3 V, where we have a sensitivity ranging from 6.2× 10^−10^ to 3.4 × 10^−9^nC/p, and the linearity at 145 MeV in the range from 8.3 ×10^7^ p/(cm^2^s) to 2.49 × 10^10^ p/(cm^2^s) is very good (R = 0.99964).Part of the work presented in this paper has been published in the proceedings of the SEIA 24 conference [24].

## Data Availability

Data are contained within the supplementary material.

## Supplementary Materials

The following supporting information can be downloaded at: https://www.mdpi.com/article/10.3390/s25041263/s1, The supplementary material includes: Data for Figure 4 and Figure 6, Figure 7, , Figure 8, Figure 9 and Figure 10 a plot of sensitivities at various tube voltages and an excerpt of the Ph.D thesis of Francesca Peverini about I/V curves normalized with sensor volume.
