# Supplementary material for: Hydrogenated Amorphous Silicon Charge-Selective Contact Devices on a Polyimide Flexible Substrate for Dosimetry and Beam Flux Measurements
Source: Sensors (Basel). 2025 Feb 19;25(4):1263. doi: 10.3390/s25041263 (PMC11860948; doi:10.3390/s25041263)
Supplement: Supplementary file 1 [file sensors-25-01263-s001.zip › sensors-3446936-supplementary/Supplementary material/PhD_Peverini_Francesca-61-78.pdf]

6 Device Results

Unlike solar cells for radiation detection, thicker diodes (in the micrometers range) are required for sufficient energy deposition in the material and a better signal-to-noise ratio. One main obstacle to achieving this objective has been the production of thick a-Si:H diodes with low defect density that can be fully depleted at relatively low bias voltage. It should be emphasized that a reasonable electric field is essential for charge collection in a-Si:H devices as carrier diffusion plays little role in charge collection. One fundamental difficulty encountered in obtaining fully depleted thick diodes lies in the nature of amorphous silicon.
Hydrogenated amorphous silicon has a high density of states in its band gap, as discussed in chapter 2 [2]. The shallow states have a dominant influence on carrier mobility as carriers are trapped and detrapped in the shallow states during their transit [54]. The deep states are crucial in determining the carrier lifetime. These states are also responsible for creating localized charge density inhomogeneities in the material which can drastically shapes the electric field.

The thickness to which the electric field penetrates in a thick a-Si:H pin diode is crucial to its usefulness as a radiation detector. High density of localized charges in the intrinsic layer implies that (when the diode is under DC bias) the electric field would no longer be uniform, unlike a crystalline pin diode in the same work conditions. The carrier mobility in hydrogenated amorphous silicon is small compared to crystalline silicon. The electron mobility is 1 cm^2/Vs, a factor of $\sim$ 1000 smaller compared to crystalline silicon. The hole mobility in this material is even smaller with a value below 0.01 cm^2/Vs. For these reasons is easy to classify amorphous silicon devices as slow in comparison with other sensors.

The main work activities, that have been carried out in this work, can be classified into 3 different macro categories:

1. Prototype assembly (the entire growing procedure is carried out at the EPFL facility and Sapienza University of Rome);

2. Performance and electrical characterizations that include (for each prototype):

    - I/V characterization for the study of the dark current behavior
    - Intrinsic current fluctuation (noise) measurements
    - Sensitivity estimation and dose-rate calibration of the sensors
    - Linearity test of the sensor response performed (if possible) on the entire dynamic range of the device

[2] If a trap is present nearer to the band edge, they are termed as shallow traps or tail; however, if they are present farther from the band edge they are termed as deep traps

3. Quality testing through spectroscopy related techniques to study structural and electrical transport properties of the material;

As described in chapter 2.4, two basic detector configurations (Fig.32) were constructed to evaluate the performances of the various prototypes.

1. pin diodes with different areas and thicknesses;

2. Charge Selective Contact (CSC) with different areas and thicknesses;

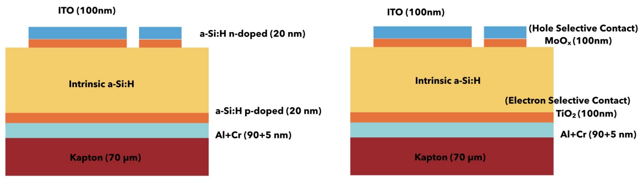

Figure 32: Cross-sectional schematic representation of the two types of devices tested.

We measured the detector response in dark conditions for different bias voltage (as leakage current estimator) to evaluate the noise level and sensor quality. Then the detector current under X-ray irradiation at different dose rates was measured. Details are discussed in section 5.2. This quantity is related to the long-term charge collection efficiency which is a useful quantity to determine the performance of devices when used in a beam monitoring application. From the linear fit of these data, we extracted the sensitivity.

6.1 Device performance in dark conditions

Characterizing a solid-state radiation detector in the absence of a signal is a critical step in evaluating its baseline performance and understanding its inherent noise levels. This process involves measuring key parameters such as leakage current and electronic noise, which significantly affect the detector's sensitivity and resolution. By analyzing the detector's behavior without any radiation input, it is possible to identify and address noise sources, ensuring that the detector can reliably distinguish actual radiation events from spurious signals. The observed noise typically arises from two primary sources:

1. Electronics-Related Noise: This includes the intrinsic noise of the acquisition electronics and current fluctuations caused by temperature variations.

2. Device Noise: This is often the dominant noise source in solid-state detectors and is influenced by the material's properties, device architecture, and operating conditions.

In detectors made from amorphous materials, such as a-Si:H studied in this thesis, additional factors must be considered, with respect to c-Si devices. Material-specific properties, like the hydrogen concentration directly influence the density of defects and, consequently, the noise levels. Moreover, every design element of the detector, like contacts, doping profiles, and structural features, affects the noise characteristics.

For all the measurements presented in this chapter, the detector is biased and readout through a source meter (Keithely 2410/2400) directly connected to the detector. A custom python software has been developed in order to allow the communication between the source meter and the user allowing the remote control of the Keithley. It gives the possibility to apply a voltage (or a sweep of voltage with a define step) and to monitor in real time the response of the sensor measuring the output current.

When the program is launched it establishes the communication with the Keithley, setting the desired voltage and simultaneously creating the plot of the current versus the acquisition time. The acquired current represents the leakage current, which is the small current that flows through a device even when no signal is present. Ideally, this current should be minimal, as it represents an undesired component that contribute to noise with its fluctuation. When the sensor is biased and the data acquisition starts we have to wait for the stabilization of the dark current, (Fig. 33 (left)) that will happen after a time varying sensor by sensor. After the stabilization the current the fluctuations of the dark current distribute according to a Gaussian whose width is a measure of the uncertainty (Fig. 33 (right)).

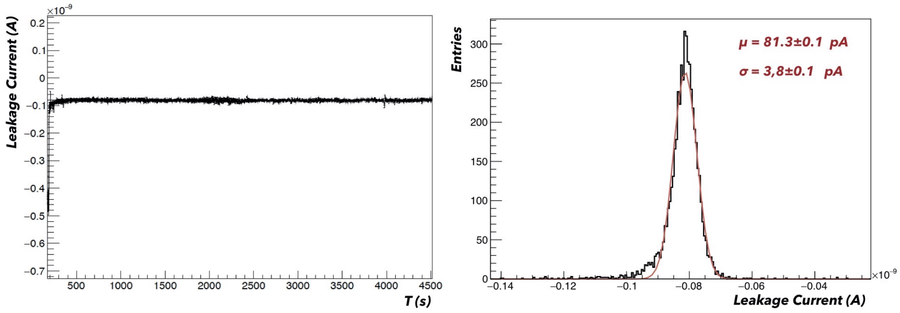

Figure 33: Current acquired by the sensor as a function of the acquisition time (on the left). Distribution of the acquired current, with a Gaussian fit overlaid, and the mean value and sigma of the fit in A (on the right).

For each value of the electric field E, the leakage current stabilize to an asymptotic value (see Fig.33). The stabilization is due to the release of excess charge trapped in metastable states in the intrinsic layer of the sensor. After the excess charge is released, the leakage current reaches a steady-state value which is

maintained by the thermal excitation of electrons from the valence band to the conduction band. The thermal excitation of electrons is typically mediated by localized states in the energy gap which arise because of defects in the a-Si:H intrinsic layer. The magnitude of this thermally generated leakage current is therefore related to the number of defects in the sensor. As shown by the trend of the leakage current over time, the sensor maintains excellent response stability. This is essential as it ensures a stable background value over time, which will later need to be subtracted during signal analysis.

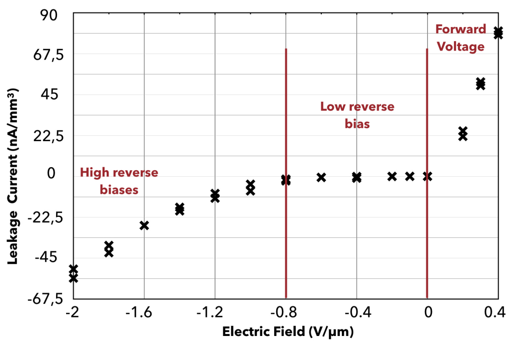

Figure 34: Typical I-V curve of an a-Si:H diode.

After this, the variation of the leakage current value with respect to the applied bias is measured (Fig.34). This is important not only to get the background level but also to identify the optimal bias value for the device operation (where depletion of the junction is maximal). Obviously, the leakage current contribution needs to be subtracted from the acquisitions in the presence of a signal. The ideal compromise we seek minimizes noise while maximizing the electric field for charge collection. To measure the I-V characteristics of the sensor the output current at fixed bias voltage for a defined time interval is processed. The mean value and sigma of the current distribution for each bias voltage are extracted as parameters from the Gaussian fit (an example of the acquisition and fit is shown in Fig.33, the applied electric field in the example shown is $E \sim 2V/\mu m$). The IV characteristics of a large number of a-Si:H devices (a total of at least thirty prototypes, each with at least four pads) have been studied. The leakage current vs bias voltage function typically shows three main regimes:

1. Low reverse bias: the leakage current increases gradually due to increased electric field and carrier drift. This part of the curve may be relatively linear.

2. Higher reverse biases: the leakage current level off and than increase sharply. Levels off, means that it reach a stable depletion region where additional bias no longer significantly affects carrier generation. In contrast, the sharp increase region indicate the onset of avalanche breakdown

in the junction, where impact ionization rapidly generates additional carriers, leading to a large increase in current.

3. Forward bias: The forward bias voltage reduces the barrier at the pn junction, allowing a significant number of charge carriers (electrons and holes) to cross the junction. This leads to an exponential increase in current.

To ensure the reliability of the detectors, we first studied the repeatability of measurements taken with the same sensor. This step was critical to verify that the sensor's characteristics remain stable over time and that its performance is consistent across multiple measurements under the same conditions. Ensuring this repeatability is essential for confirming the long-term reliability of the device and for eliminating potential variations due to temporal instabilities.

In the Fig.35, 16 IV curves obtained with one of the CSC-type sensors deposited on Kapton are shown. The results indicate that the variation between the repeated measurements ranges from 9% to 1%. Notably, the variation tends to be smaller in the reverse bias region, where the leakage current remains constant. This reverse bias region is particularly important, as it corresponds to the optimal bias value for the sensor's proper functioning, where the sensor operates most reliably and with minimal noise.

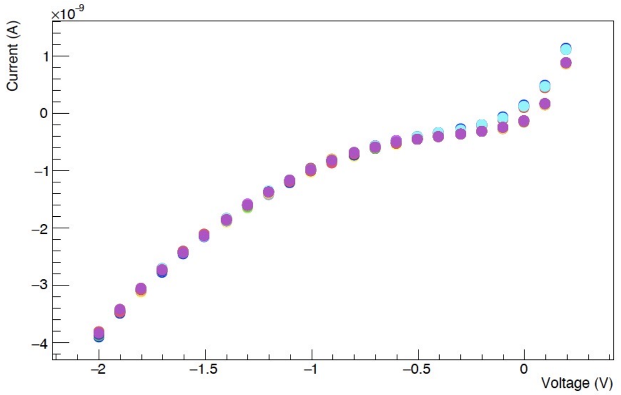

Figure 35: 16 repetitions of the I-V curve for one of the sensors under study.

Once the leakage current (as a function of bias) is measured, the next step is to evaluate its fluctuation over time. This is essential because, although the absolute leakage current value can be subtracted from each measurement to isolate it from the signal, every temporal trend directly alters the output signal. The minimum signal that the sensor can reliably detect, as well as the smallest distinction between two different signals that the sensor can identify as separate is affected by this lack of time-stability. In Fig.36, some of the noise values obtained with different sensors under the same applied electric field are

shown.

After confirming the repeatability of a single sensor, we extended the study to test the uniformity among multiple sensors fabricated with identical geometric characteristics and deposition substrates. The leakage current behavior was measured across these sensors to evaluate whether their responses were consistent and reliable. This step aimed to verify the stability of the fabrication process and the uniformity of the sensors' performances.

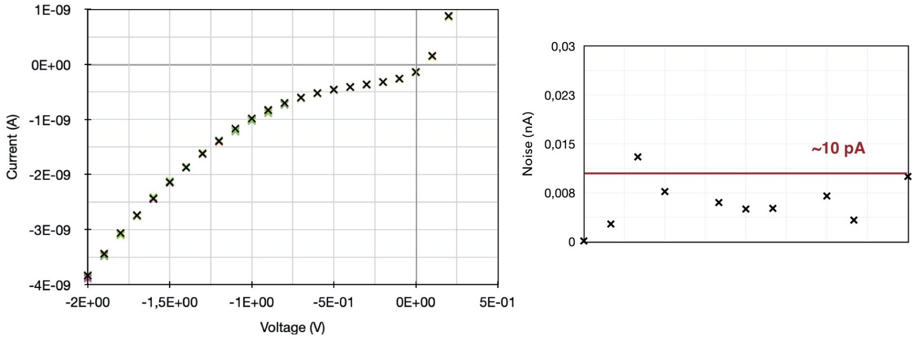

Figure 36: IV curve for 15 pin sensors on Kapton (left), calculated value of leakage current fluctuation for the different sensors (right).

In Fig.36, the results for 15 different sensors belonging to the same production batch are shown, highlighting the minimal variation in leakage current measurements. The high degree of uniformity observed underscores the robustness of the fabrication process and confirms that these detectors are capable of delivering consistent, repeatable, and reliable measurements for practical applications.

After evaluating the reproducibility of the IV response of the sensor and the uniformity of this response across various detectors built with the same characteristics, the next step is to test how the IV curve changes as a function of:

1. Substrate (c-Si and Kapton)
2. Contact (CSC and pin)
3. Production Batch
4. Geometry (active volume)

To investigate this, the IV characterization was performed for different sensor type, and the responses were compared (see Fig.37) after normalizing the current response to the sensitive volume and electric field. This allowed for a better

understanding of how the detector's design parameters affect its electrical characteristics and performance. It should be noted that this type of comparison is not straightforward, as there are many parameters that need to be studied and can vary, and not all of them can be easily controlled, especially deposition-related parameters.

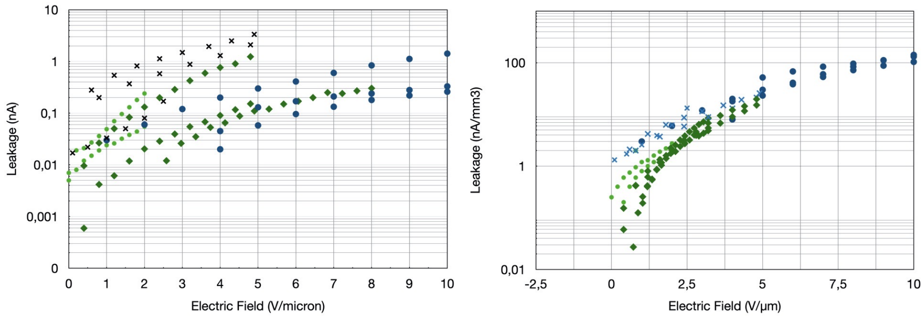

Figure 37: IV curves of different sensors without any normalization (on left). IV curves with the current response normalized to the sensitive volume of each sensor (on right).

As observed, the detector response does not fundamentally depend on the active volume of the device. When the signal is normalized, to the active volume (assuming all other characteristics remain constant), their responses are comparable.
Specifically, the data points marked with blue "x" represent CSC-type sensors deposited on c-Si, while blue circles correspond to pin-type sensors deposited on c-Si. The green diamonds represent pin-type sensors, and the green circles correspond to CSC-type deposited on Kapton. The graph does not reveal a significant dependence on the type of contact used. However, a noticeable distinction emerges between devices fabricated on different substrates. In particular, devices deposited on Kapton exhibit a less stable leakage current, especially at low electric field values.

It is worth emphasizing that the devices included in this analysis were produced across multiple fabrication batches. Since the detector response is highly sensitive to deposition parameters, such as those influencing the defect density, it is inherently challenging to achieve a perfectly uniform response across all sensors of this type. This consideration is supported by the fact that when devices from the same wafer are compared, the overlap in their responses is very high (see Fig.36).

In conclusion, the sensors characterized in this thesis exhibit uniform, repeatable, and stable performance over time in the absence of an external signal. The dark current is observed to scale proportionally with the sensitive volume of the sensor, and no significant differences in response are attributable to the

type of contact used. Furthermore, leakage current fluctuations are consistently maintained below 10 pA in the majority of cases, with a maximum deviation of under 30 pA even in less favorable conditions.
This low level of noise is of critical importance as it facilitates the detection of low-intensity radiation and small signal variations, thereby enhancing the detector's sensitivity. Moreover, a low noise contributes to a higher Signal-to-Noise Ratio (SNR), which is essential for achieving accurate and reliable measurements, further underscoring the suitability of these sensors for precision applications.

Finally these measurements also allow us to determine the optimal voltage for operating a sensor based on its IV curve. The best voltage for operation is typically chosen within the saturation region, where the current has stabilized, and the sensor operates with maximum efficiency. This ensures that the active layer is fully depleted, charge collection is optimized, and noise is minimized. The voltage should also remain well below the breakdown threshold to avoid damage and ensure stable long-term performance. In the case of amorphous silicon (a-Si:H) an electric field as high as possible is often needed to overcome trapping and ensure efficient charge collection, given all these considerations, an electric field value of $\sim 2\ V/\mu m$.

6.2 X-ray device characterization

The sensors were initially tested with an X-ray tube available in the INFN laboratory of the Perugia section. This first characterization phase aims to first verify the linearity of the detectors response to X-rays to quantify the stability of the signal, its repeatability and finally to obtain a measure of sensitivity and signal-to-noise ratio. The sensitivity of a detector to ionizing radiation is a critical factor that determines, together with the noise level, its ability to accurately detect and measure radiation events, even the small signal ones. Sensitivity refers to how effectively the detector can register the energy deposited by ionizing charged particles or photons and convert that energy into a measurable signal. Sensitivity indicates also the device's capability to distinguish between two signals that are very close, ensuring their clear separation and accurate recognition even under challenging conditions.

The X-ray tube operates at a range of voltages, typically between 10 kV and 50 kV, allowing for flexibility in applications that require different energy spectra. The current setting can be in the range 5 μA to 200 μA, with a 4 W power limit, depending on the configuration.
In general, an X-ray tube like the one used for the measurements shown provides photons with an energy that is partly specific to the tube but also distributes over a broad continuous spectrum (see Fig.38). Hence, the total X-ray spectrum is a combination of both the continuous and characteristic spectra, resulting in a broad distribution with superimposed sharp peaks. The continuous spectrum, is generated by bremsstrahlung emission when high-energy electrons are decel-

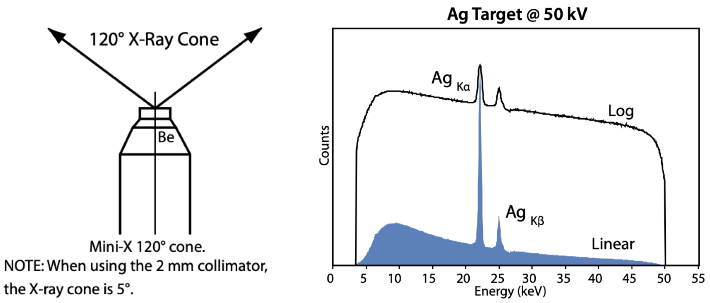

Figure 38: MiniX X-ray tube: mechanical dimensions and spectrum for Ag target [55].

erated upon striking the anode, which is typically made of tungsten. As these electrons lose energy, they emit X-ray photons across a broad range of energies, forming a curve that generally peaks at lower energies (about a third of the maximum energy of the colliding electrons) and tapers off at higher energies until it reaches the endpoint identified by the maximum possible energy, corresponding to the electron energy given by the tube voltage.
With this X-ray tube we obtain an approximate flux of about 10^6 counts Hz/mm^2 on the X-ray cone axis at a distance of $30cm$ (setting the voltage and current to the values of 50 kV - 1 μA), [55]. Starting from this value, increasing the tube current proportionally increases the number of electrons striking the anode, leading to greater intensity in both spectra.

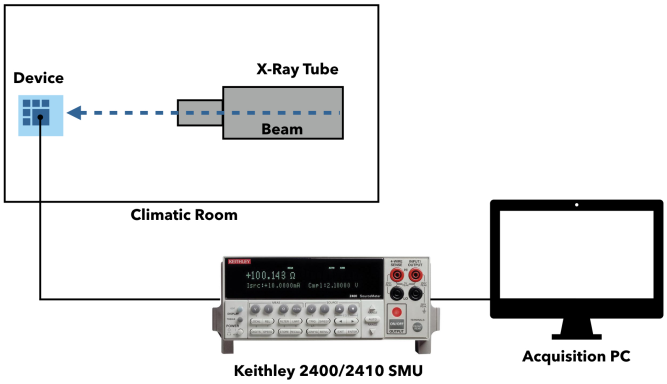

Figure 39: Diagram of the setup used for the X-ray tube acquisitions conducted in the INFN laboratory of the Perugia Section.

The experimental setup used for the various measurements is shown in Fig.39. The detectors are glued and bonded to a printed circuit board PCB frame, which is connected to an interface board linked to a Keithley 2400 for biasing the sensor and measuring the output current with a resolution of 1 pA.

The sensors are exposed to the X-ray tube described above. During the characterizations with sources, two parameters are varied to study the device's response changes in relation to them: the applied electric field and the tube current. In this way, we can study how the detector's response changes with respect to the beam intensity, and how (or if) the gain varies with the increase of the applied bias voltage.

The tube voltage is kept constant to avoid changing in the photon energy spectrum and to study the response solely as a function of the photon flux. For the same reason, the distance from the tube is also kept fixed; otherwise, a different thickness of air traveled by the photons before reaching the detector would alter the energy spectrum and also the different distance would alter the photon flux.

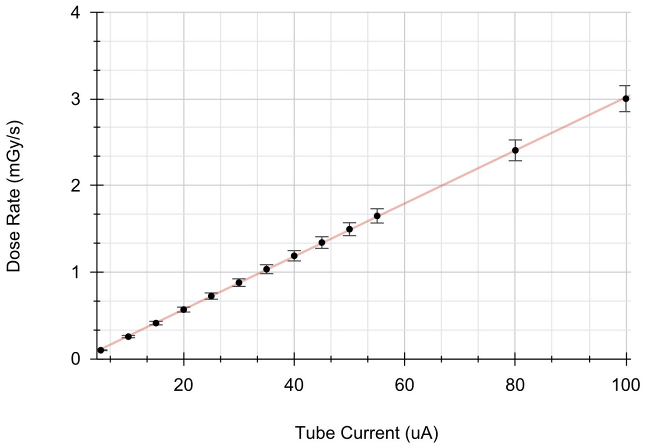

Figure 40: example of a calibration curve with the X-ray tube set at 40 kV. The line represents the dose rate reading of the dosimeter as a function of the tube current.

The dose rate of the emitted radiation has been measured using the Cobia Flex dosimeter probe, [56], at many positions in the measurement box, mapping the [tube voltage, tube current] pairs in the whole parameter domain. In this way we could put in correspondence the X-ray flux generated by the current in the X-ray tube and the signal measured by the sample placed in the same position, using the Cobia calibration values (Fig.40).

The protocol used for all measurements requires a sequence of $Beam_{OFF}$ / $Beam_{ON}$ / $Beam_{OFF}$ to allow each time the determination of the leakage current and the related noise level.

An example of an acquisition with ionizing radiation exposure is shown in Fig.41, which displays the variation of the acquired detector current as a function of the acquisition time.

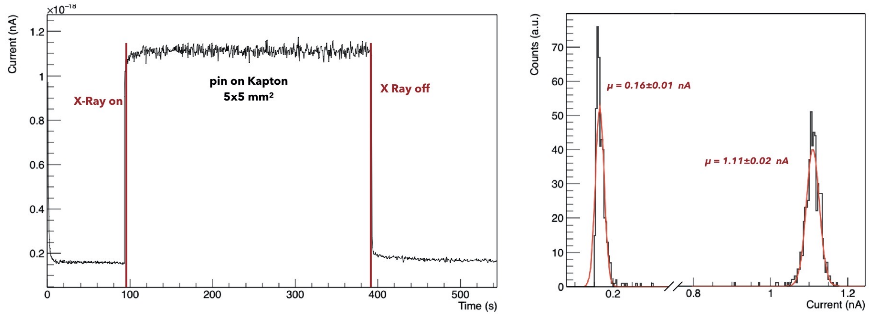

Figure 41: On left: signal and leakage distribution with corresponding gaussian fit. On right: time profiles of the current measured (in this example, the sensor is set to 2 $V/\mu m$).

The graph on the left clearly illustrates the rising and falling edges of the signal, corresponding to the activation and deactivation of the X-ray beam. It is noteworthy that the time required for the signal to stabilize is comparable to the time it takes for the X-ray tube to reach the preset voltage and current levels, which is approximately 10 seconds. This observation is crucial as it highlights the consistency between the sensor's response time and the operational dynamics of the X-ray source.
The graph on the right displays the distribution of the acquired sensor current. Two distinct peaks can be observed: one corresponding to the leakage current and the other to the signal generated when the X-ray beam is on. These distributions are clearly distinguishable, demonstrating that the signal significantly surpasses the noise level.

To analyze the data quantitatively, a Gaussian fit is applied to both distributions, enabling the extraction of their mean values and standard deviations. These parameters are fundamental for determining the sensor's reading under the specific dose rate applied during the measurement. We can also extract a SNR value of 42, which is defined as follows:

$$SNR = \frac{\mu_S - \mu_N}{\sigma_{TOT}} \tag{18}$$

$$\sigma_{TOT} = \sqrt{\sigma_S^2 + \sigma_N^2} \tag{19}$$

This methodology allows for the systematic reconstruction of the sensor's response as a function of the dose rate. An example of this behavior is presented in Fig. 42, where the relationship between the sensor signal and the dose rate is modeled using a linear function of the form:

$$I = k \cdot D_r + I_0$$

where I is the output current, k is the sensitivity (expressed in nA/(cGy/s)), D_r is the dose rate and I_0 is a possible current offset due to the setup.

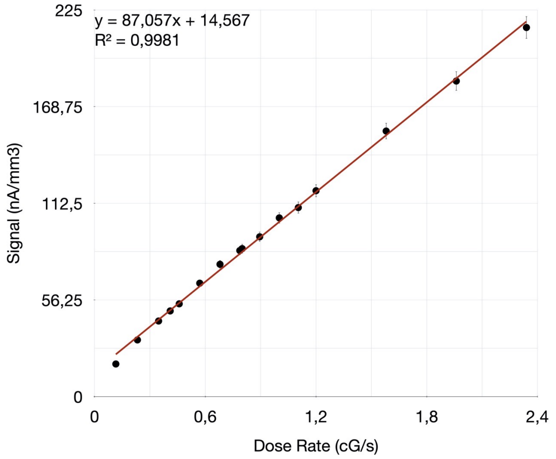

Figure 42: Current response of the amorphous sensor (pin diode on Kapton) as a function of the dose rate; the red line represents the implemented linear fit (the reported error bars represent approximately 3% fluctuation).

This analysis and fitting procedure was systematically repeated for each of the sensors tested throughout the course of this thesis work, ensuring comprehensive evaluation and consistency across the devices. The results consistently demonstrate that the detector's response to the applied stimulus is approximately linear, with an average value of the R^2 coefficient very close to 1 ($R^2_{Mean} = 0.997$). The excellent linearity in the response is an outstanding result, as it allows the use of a single calibration factor for the sensor, without the need for additional corrections, at least within the studied dose rate range.

Furthermore, the SNR was calculated for each sensor, to provide a quantitative idea of the ability to distinguish the signal. It is useful to report the SNR value obtained in the most challenging case, i.e., the measurement with the lowest tested signal. In this case, with a signal of only $0.0000295 Gy/s \sim 30$ $\mu Gy/s$, a SNR value of 2.2 is achieved. This S/N ratio highlights the sensor's capability to detect low-dose signals effectively while maintaining measurement accuracy and reliability.

6.2.1 Long term stability and repeatability of the signal

In this paragraph the procedures used to determine the stability of the devices during time are described. The characterizations conducted in this work typically involve short acquisition durations, approximately 2 minutes, which are insufficient to test the long-term stability of the signal. As previously mentioned, one critical parameter to evaluate is the stability of the signal over time, which is essential for demonstrating the feasibility of these sensors for prolonged exposure measurements. A stable sensor will exhibit minimal drift, fluctuations, or degradation in its signal under prolonged exposure to a fixed input.

First, the stability of the leakage current in the absence of any signal was verified. As shown in the graph, the sensor's background over nearly 12

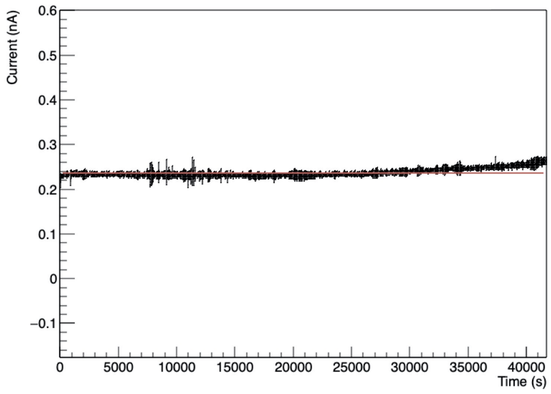

Figure 43: Time profiles of the leakage current measured. Red line represent the mean.

hours of operation did not change significantly. The leakage current starts at a value of 0.233 nA and reaches 0.261 nA by the end of the acquisition. Throughout the acquisition, the noise remains stable at 0.003 nA. In particular, to be quantitative, during the first 8.5 hours of operation, the percentage variation from the mean (represented by the red overlaid line) is 3 %. The increase up to 11.4 % of the absolute value of the current after the initial 8.5 hours is most likely due to a temperature rise, which occurs after many hours of continuous operation despite the setup being placed in an isolated chamber. The temperature increase is at most about 1 degree per hour of continuous operation

Subsequently, the stability of the sensor's response under stimulus was also studied. To address this, dedicated measurements were performed in which the sensor was subjected to a constant stimulus for an extended period of time, while the output signal was continuously recorded. An example of such an acquisition, lasting approximately 6 hours, is shown in Fig. 44.

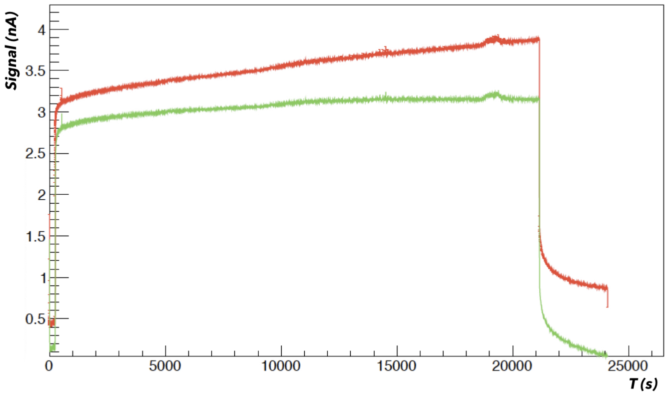

Figure 44: Time profiles of the current measured. Red line represent the raw data, green line represent the data after exponential background subtraction.

As shown in the figure, two temporal profiles are presented. The red profile represents the raw data acquired during the measurement, while the green profile corresponds to data corrected through the subtraction of an exponential background. This step was necessary to isolate the true behavior of the detector's response over time.
The correction addresses an observed phenomenon during the extended duration of the measurement. Specifically, the temperature inside the measurement enclosure gradually increased over time. This rise in temperature, likely due to environmental factors and the operational heat dissipation of the equipment, led to a corresponding change in the leakage current of the sensor.

The leakage current, due to its nature, is highly sensitive to temperature variations. These processes typically follow an exponential temperature dependence, causing the leakage current to increase non-linearly as the temperature rises. Without accounting for this background effect, it would be challenging to determine whether the observed changes in the measured current were due to the detector's inherent stability or the external temperature variation. To mitigate this influence, an exponential function was fitted to the temporal trend of the leakage current and subsequently subtracted from the raw data. This approach effectively removed the contribution of temperature-induced fluctuations, allowing for a clearer evaluation of the detector's actual performance over time.
The fit was, off course, performed by excluding the data acquired during the activation of the beam, using only the data recorded in the absence of the signal. This approach ensures that any potential drift in the signal, which is the subject of our study, is not inadvertently removed during the background correction process.

By isolating the leakage current behavior in the absence of irradiation, the correction accurately addresses temperature-induced variations without compromising the integrity of the detector's response under constant stimulus conditions. After applying this correction, the results revealed that the corrected current showed an 8% variation over 7 hours of continuous operation. This residual increase could also be due to the rising temperature of the X-ray tube that influence the photon flux emitted toward the exterior world. This level of stability highlights the robustness of the detector's response and its potential suitability for long-term measurement applications, provided that temperature effects are appropriately managed.

Another essential characteristic to study is the repeatability of the measurements, as we aim to ensure that the sensor's response is reliable and consistent. To evaluate this, multiple consecutive acquisitions were performed while maintaining a constant dose rate from the X-ray tube, an example is shown in Fig.45.

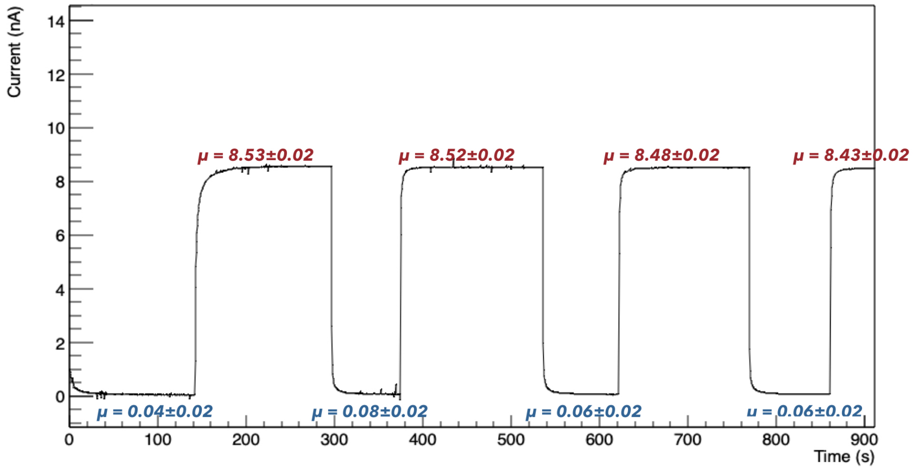

Figure 45: Time profiles of the measured current: the acquisition includes repeated measurements of the sensor's response under the same stimulus (40kV-100μA from the X-ray tube). The image shows the average value and the standard deviation of each acquisition segment, both in the dark and during emission.

As seen, by comparing the average values of the signal acquired under the same stimulus, the sensor provides the same current response, with a percentage variation from the mean under 1%.

6.2.2 Sensitivity

The final part of this characterization work focused on measure the device's sensitivity and comparing the response of various sensors based on their construction parameters: area, thickness, and type of contact.

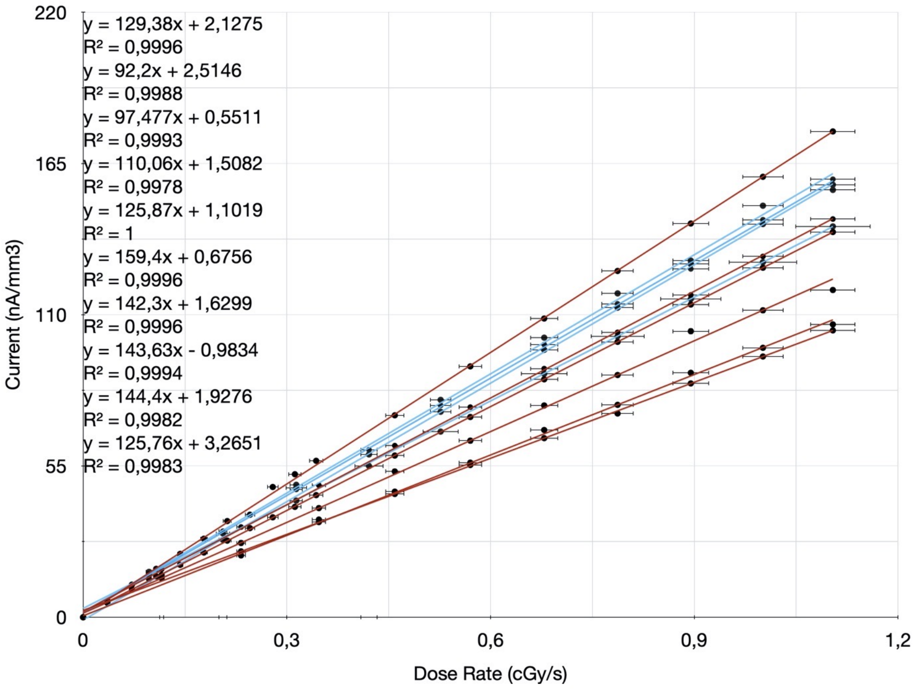

Figure 46: Comparison of the experimental sensitivity (normalized to volume) measured on 10 sensors: 6 pin-type sensors (red line) and 4 CSC-type sensors (blue line).

The graph in Fig.46 displays response curves for a selection of sensors used in this study, reported as an example. Specifically, the responses of 10 sensors deposited on Kapton with two different sensitive volumes are shown. Similar to the leakage current behavior, the detector response scales with the sensitive volume, provided the substrate and contact type remain consistent. It should be noted that the difference between the various lines, which are not entirely overlapping, also depends on the fact that the tested sensors do not always belong to the same production batch.

Once the sensitivity for each tested sensor was measured, of which Fig.46 represents only a small portion, an average sensitivity value was extracted for each type (with same substrate, contact type, and sensitive volume), all shown in the table 3. All sensitivities are normalized to the sensitive volume and

measured under the same applied electric field ($\sim 2V/\mu m$), enabling a direct comparison between different sensor configurations. The table reports the extracted sensitivity values for various detector configurations. As shown in the normalized values in the third column, we observe consistent sensitivity across all detectors. The mean sensitivity values are 97 ± 5 $nC/cGy\ mm^{-3}$ for pin detectors on Kapton, 162 ± 17 $nC/cGy\ mm^{-3}$ for CSC detectors on Kapton.

Table 3: Average sensitivity measured for each type of sensor tested.

X-ray Sensitivity		
Type of Sensor	Volume (mm^3)	Normalized Sensitivity ($nC/cGy\ mm^3$)
pin on Kapton	0.06	105 ± 13
pin on Kapton	0.01	92 ± 2
pin on Kapton	0.125	94 ± 2
pin on Kapton	0.02	117 ± 1
CSC on Kapton	0.125	145 ± 1
CSC on Kapton	0.02	178 ± 1
pin on c-Si	0.01	903 ± 40
pin on c-Si	0.0025	1040 ± 40

The sensitivity values of the devices deposited on c-Si are reported, as previously mentioned in Chapter 2, as a reference, since this is the first time devices deposited on a substrate such as Kapton have been characterized. We expect that the differences are primarily due to the characteristics of a-Si:H and, to some extent, to its adaptation to the deposition substrate. In general, Polyimide substrates (Kapton) exhibit defects (trenches, holes, bumps) that can lead to a significant decrease in the performance. Chapter 7 will present a detailed spectroscopic investigation to explore also the reasons behind this result.

The sensitivity study presented so far was conducted maintaining a fixed electric field at the predefined value of 2 $V/\mu m$. However, the possibility of using these devices without external power supply has also been explored. This is particularly advantageous for medical applications, where the ability to operate without an external power supply offers several key benefits, as the development of compact and portable devices and reduces electrical risks. Unfortunately, completely eliminating the need for an external power supply was only possible for CSC-type sensors. Actually, pin-type sensors, when not powered, are unable to direct the current flow in a preferred direction. This can be observed as the measured current during signal presence may have the opposite polarity compared to what is expected. The possibility of using CSC-type sensors without external power supply can be explained by

considering that, by design, CSC sensors typically rely on the properties of the material used as contacts (such as mobility) to generate or direct the flow of charge when exposed to stimuli. Therefore, they can function effectively even in the absence of external voltage. Pin type sensors on the other hand, when not powered, lack the necessary electric field to drive the charge carriers in a controlled direction.

However, even though pin sensors cannot be used without external voltage, it has been demonstrated that they are still able to function with a very low electric field. Below it is shown the response of 8 pin sensors to an applied electric field of only 0.5 $V/\mu m$. This result was particularly achievable for very thin devices. The graphs shown in the figure refer specifically to devices with a thickness of only 0.4 μm. The sensors tested in this configuration achieve a sensitivity of: 64 $nC/cGy\ mm^{-3}$.

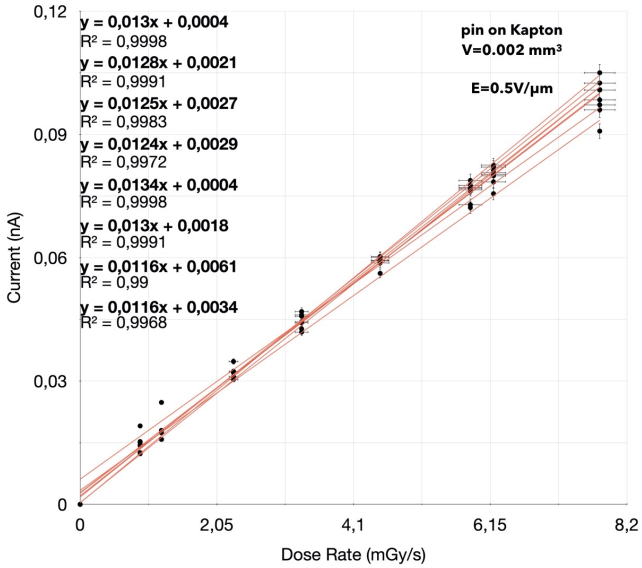

Figure 47: Comparison of the experimental sensitivity measured on 8 pin-type sensors, belonging to the same array.

6.2.3 Mapping and characterization of the charge collection

To design a device suitable for dosimetry and beam monitoring purposes it is also very important to have a detector with a well-defined sensitive volume. This is because the presence of possible lateral diffusion currents in the substrate can create distortion during beam reconstruction and alter dose evaluation.
