# Supplementary material for: Hydrogenated Amorphous Silicon Charge-Selective Contact Devices on a Polyimide Flexible Substrate for Dosimetry and Beam Flux Measurements
Source: Sensors (Basel). 2025 Feb 19;25(4):1263. doi: 10.3390/s25041263 (PMC11860948; doi:10.3390/s25041263)
Supplement: Supplementary file 1 [file sensors-25-01263-s001.zip › sensors-3446936-supplementary/Supplementary material/Sensitivities at various voltages.pdf]

Photocurrent vs tube current (or dose rates) at various voltages.

The first graph shows the photocurrent versus tube current at various voltages;

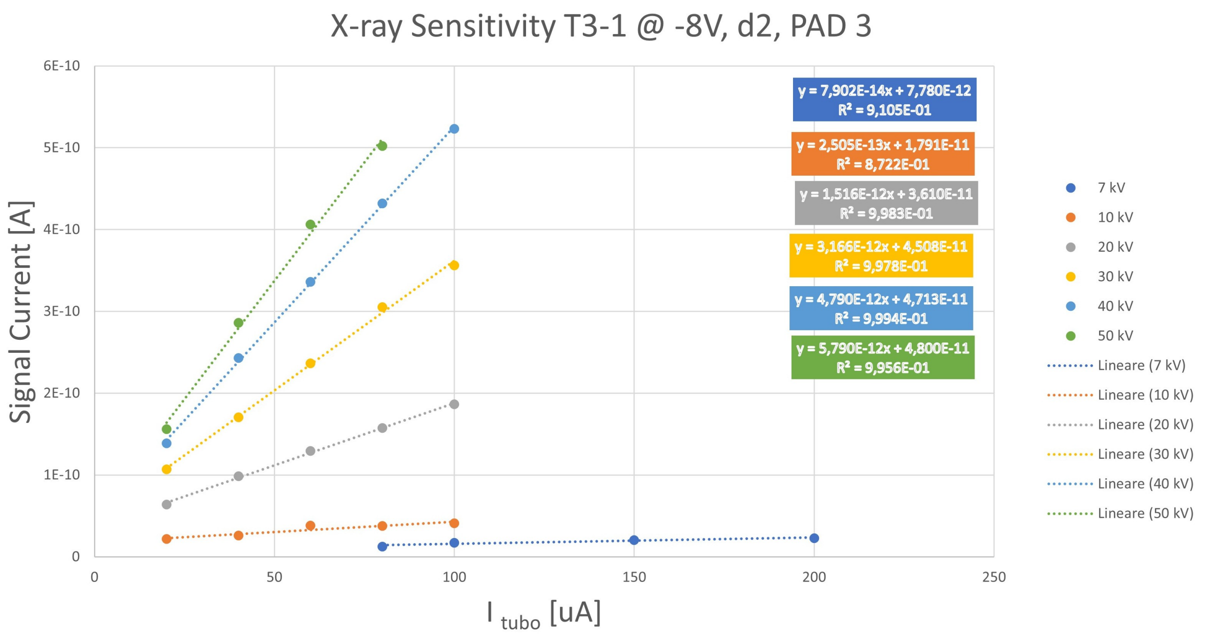

from this graph it is possible to notice that the sensitivity compared to the various tube currents (directly related to photon fluxes) increases with tube voltage (related to photon energy).

Graph of Signal current versus total dose at various tube voltages

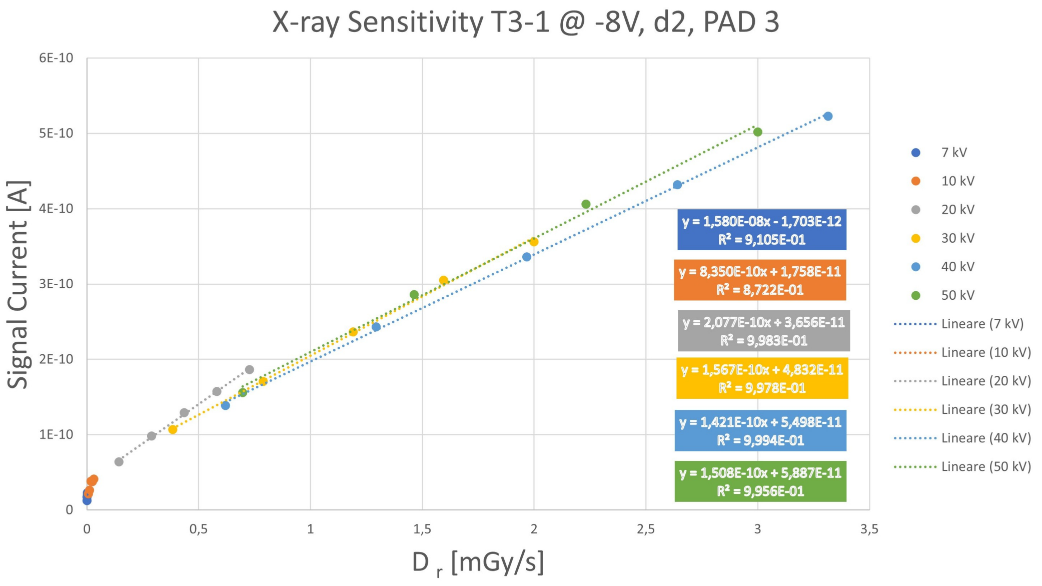

In this case except for the lowest tube voltages where the dose is very small the various lines almost overlap.

Calibration curve Dose rate versus tube current at various tube voltages.

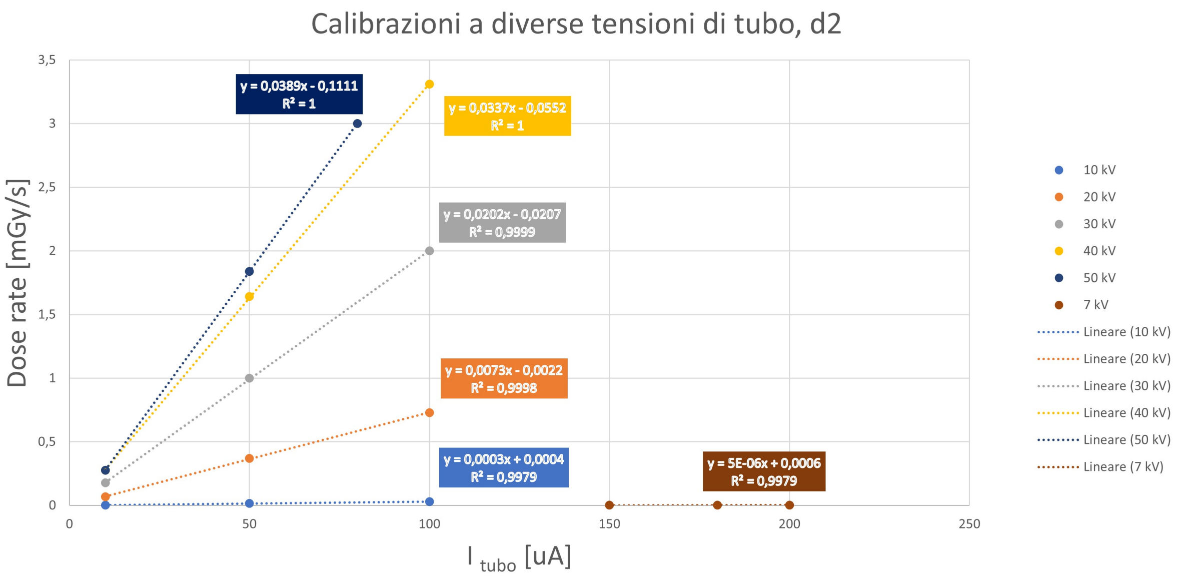

Calibration coefficients for the conversion tube current -> dose rate at high tube voltages smaller current are needed to generate a given dose and this compensate the higher increase of slope at various tube voltages in the first graph compared to the second graph.
